# Apples and oranges in HEMS – Do we know what we compare?

**DOI:** 10.1186/1757-7241-23-S2-A29

**Published:** 2015-09-11

**Authors:** Kristen Rasmussen, Stephen JM Sollid

**Affiliations:** 1 Research Department, Norwegian Air Ambulance Foundation, Drøbak, Norway; 2 Network for Medical Sciences, University of Stavanger, Stavanger, Norway; 3 Møre and Romsdal Health Trust, Ålesund, Norway

## Background

The choice of medical staffing in helicopter emergency systems (HEMS) is continuously under debate. Many studies have attempted to demonstrate the benefit of one profession over the other. Only one RCT-study on staffing models in HEMS randomized patient care to either a physician and nurse or nurse and paramedic [1]. None of the studies have however succeeded in providing the answer to what is the optimal medical crew concept. One of the challenges in comparing the effect of different professions on outcome is the lack of a precise definition of competence of each profession involved in HEMS.

The HEMS Medical Crew Survey was designed to collect data on different HEMS crew compositions and the rationale behind them. We here present data from this survey describing the diversity of formal competence for physicians, nurses and paramedics that are part of the regular crew in HEMS.

## Method

Medical directors of HEMS-services in Europe, North America, Australia, New Zealand and Japan were invited to complete a web-based questionnaire (SurveyXact^TM^). The survey was open between June 1st and October 15^th^, 2014. All respondents were blinded to the researcher. The study was approved by the Data Protection Official in Norway and exempted from ethical approval.

## Results

The survey received 111 submissions. Forty-four submissions did not contain sufficient data regarding crew and were excluded. The remaining 67 submissions reported data from 18 different countries.

### Physicians

Physicians were part of the medical crew in 73.1% of the HEMS services. The most common specialty for physcians was anaesthesiology (85.7%), followed by emergence medicine (59.2%), surgery (22.4%), internal medicine (20.4%) and a range of other specialties (16.3%). Some service had physicians with different specialities. Only board certified physicians were allowed to work in 63.3% of the services, 34.7% used both certified specialists and physicians in training.

### Nurses

The survey defined Registered Nurses (RN) as nurses with education corresponding a Bachelor's Degree, and certified nurses as RN with an additional exam to be certified.

RNs without additional education were part of the crew in 20.0% of the responding HEMS. Crews were staffed by Certified Flight Nurses in 57.1%, Certified Emergency Nurses (CEN) in 51.4%, Certified Critical Care Nurses in 37.1%, Intensive Care Nurses in 28.6%, Certified Transport Nurses in 25.7%, Nurse Anaesthetists in 14.3%, Neonatal Nurses in 11.4%, Certified Paediatric Nurses in 5.7% and Acute Care Nurse Practitioners in 5.7%.

### Paramedics

The titles “paramedic” and “Emergency Medical Technician” (EMT) are poorly defined and based on many different educational models. For the purpose of the survey we categorized these professional groups according their airway skills: (1) ”Only supraglottic airway device” (14.8% in this survey), (2) ”Endotracheal intubation but not rapid sequence induction (RSI)” (25.9%), (3) ”Endotracheal intubation including RSI” (59.3%) and (4) ”May use mechanical ventilator” (59.3%).

### Hems Crew Members

A HEMS Crew Member (HCM) is defined as medical professional that also acts as pilot assistant and often rescue specialist. Their medical training is EMT, paramedic or nurse. In this study the HCM had background as EMT/paramedic with only “supraglottic airway skills” in 38.5% of the services, with “intubation skills” in 38.5% and “RSI-skills” 26.9%. In 26.9% the services used RN as HCM, 15.4% used Nurse Anaesthetists and 7.7% nurse with CEN certification.

## Conclusion/discussion

Because we could not get access to databases of the medical directors in all the countries surveyed we cannot evaluate the response rate of our survey. This implies that our results cannot be fully representative of the countries surveyed.

This study shows the variety of educations and level of competence in the medical crews in HEMS. For future research on staffing models a more precise definition and description of competence of all professions is needed. Using airway skills is one way to do this for paramedics and emergency medical technicians.
